# Insights into the diversity and conservation of the chB6 alloantigen

**DOI:** 10.3389/fimmu.2025.1547896

**Published:** 2025-02-20

**Authors:** Phillip E. Funk

**Affiliations:** Department of Biological Sciences, DePaul University, Chicago, IL, United States

**Keywords:** chick (gallus gallus), B lymphocyte (aka B cell), apoptosis, CD2 family, orthologs and paralogs

## Abstract

The bursa of Fabricius has been a durable model of B lymphocyte development. Yet there are unique aspects of B- lymphocyte development in the bursa that remain to be elucidated, and these may reveal important functional differences in the avian system and distinct evolutionary mechanisms from the canonical murine and human models of B- lymphocyte development. Our laboratory has been interested in the function of the chB6 alloantigen. ChB6 has three defined alleles and is present on B lymphocytes in chicken from their earliest development at ED 12. ChB6 continues to be expressed through B- lymphocyte ontogeny as well as on a subset of macrophages. We have shown that chB6 ligation by antibody leads to rapid apoptosis. Transfection of cDNA- encoding chB6 replicates this in mammalians cells, suggesting a common signaling pathway, but there remain no clear mammalian homologues. Structurally, the extracellular domain of chB6 is similar to mammalian SLAM (signaling lymphocyte activation molecules) proteins and chB6 partitions into lipid rafts in close proximity to the B- cell receptor. The lack of homology within the intracellular domain remains puzzling. Utilizing genomic resources, we have found a number of similar molecules in both birds and reptiles; however, they show greater conservation in the intracellular domain, including an SH3 motif that we have shown to be critical in inducing apoptosis.

## Introduction

In addition to its widely acknowledged agricultural importance, the chicken is one of the foundational models of immunity, establishing the fundamental roles of T and B lymphocytes (1). Despite this, recent work on lymphocyte development has focused more on mouse and human as model systems. Nevertheless, there is appreciation for the diversity of immune models, in particular the gut as a unique site driving immune development in a number of animal species (2). Indeed, mucosal immunity has provided a window into tolerance mechanisms relevant to chronic disease. Furthermore, the divergence of birds from reptiles around 150 million years ago (3) offers the opportunity to ask about distinct solutions to basic immunologic questions, so the chicken remains a vital model of immune function.

In the chicken, B- cell precursors arise around day 12 of embryonic development (ED) and undergo V(D)J gene rearrangement of immunoglobulin loci in the embryonic spleen (4, 5). These cells then migrate to the bursa of Fabricius around ED 15 where they populate bursal follicles and begin to proliferate and undergo Ig diversification by gene conversion (6). Around the time of hatching, B lymphocytes begin to leave the bursa, seeding the secondary lymphoid tissues (7). These cells have diverse Ig and participate in immune responses. Notably, only approximately 5% of the B cells produced in the bursal ever leave there, the remainder dying by apoptosis (7). This mirrors the estimated loss of B cells during development in the bone marrow (8). In both mammals and birds, it is believed that a factor in this cell loss is the enforcement of central tolerance, removing B lymphocytes bearing autoreactive Ig specificity.

ChB6 (formerly BU-1) was identified as an alloantigenic marker of the earliest B- cell precursors and continuing to be expressed throughout B cell ontogeny. It is also expressed on a subset of macrophages (9, 10). Upon cloning the chB6 cDNA, it was described as a type I transmembrane protein of unique structure and heavily glycosylated (11). Subsequent work showed that cross-linking chB6 by antibody resulted in a rapid apoptosis (12). Furthermore, this could be replicated in mammalian transfectants expressing chB6 and regulated by anti-apoptotic proteins such as bcl-xL, suggesting a connection to a highly conserved intracellular machinery (13, 14). Later work showed that chB6 colocalizes to lipid rafts in close proximity to the B- cell receptor (BCR) and that an SH3 motif in the cytoplasmic domain was critical in mediating pro-apoptotic signals (15, 16). ChB6 was also placed among the CD2/SLAM family of lymphocyte signaling molecules based on the presence of 2 extracellular Ig-like domains. However, little homology was seen between chB6 and other SLAM members in the intracellular domain and many questions remain about how chB6 initiates apoptosis within cells and the evolution of this unique molecule.

## Materials and methods

### Cell culture conditions

DT40 cells were maintained in DT40 media cultured in DMEM (Life Technologies, Grand Island, NY), supplemented with 12% newborn bovine serum, 6% Tryptose Phosphate, 4% chicken serum, 10 mM HEPES, pH 7.3, L-glutamine, and antibiotics. BK3A cells were maintained in BK3A media cultured in DMEM, supplemented with 6% Tryptose Phosphate, 4% NBS, 1% chicken serum, 1% 1 M HEPES, pH 7.3, L-glutamine, and antibiotics. Cells were incubated at 37°C with 5% CO_2_ and passaged at a 1:10 ratio every 2 to 3 days.

### Anti- phosphoprotein western blots

DT40 cells were cultured at room temperature at 5 × 10^6^ per mL in DT40 medium with addition of either 1 μ L FU5-11G2 (anti chB6.2, ascites) (17) or 5 μ L anti chicken IgG heavy- and light- chain antibodies (Pierce Chemical Company, Rockford, IL). At the times indicated, cells were immersed in an ice water bath until all trials were complete. The cells were then centrifuged at 14,000 rpm for 5 min at 4°C. Cell pellets were lysed with 100 μL lysis buffer containing 5% 1 M Tris, 3% 5 M NaCl, 0.1% SDS, 1% Triton X, 0.5% deoxycholate, 0.2% 0.5 M EDTA, 1 mini protease inhibitor cocktail tablet (Roche, Indianapolis, IN), and 100 μL phosphatase inhibitor cocktail 2 (Sigma, St. Louis, MO) per 10 mL buffer. Cell lysates were incubated on ice for 30 min and then centrifuged at 14,000 rpm for 30 min at 4°C to remove cellular and nuclear debris. Aliquots of the supernatant were assayed for protein concentration (BCA Protein Assay Kit, Pierce). Volumes equivalent to 25 μg of protein were added to equal amounts of Laemmli buffer and boiled for 5 min and run out on 10% SDS-PAGE gels. Proteins were transferred to supported nitrocellulose membranes (Osmonics, Westborough, MA) and blocked for 2 h in 5% milk protein in 0.1% TBS-Tween. Membranes were washed 3× in 0.2% TBS-Tween and then probed with anti-phosphotyrosine, anti-phosphoserine, or anti-phosphothreonine (Chemi-Con, Temecula, CA) at 1:10,000 in 5% milk protein 0.1% TBS-Tween for 2 h. Membranes were again washed 3× in 0.2% TBS-Tween. Membranes were incubated for 60 min in anti-rabbit IgG- horseradish peroxidase conjugated secondary antibody (Boehringer Mannheim, Indianapolis, IN) at 1:2,000 in 5% milk protein 0.1% TBS-Tween and visualized by addition of CN/DAB colorimetric reagent (Pierce) or SuperSignal Chemiluminescent substrate (Pierce).

### GST pull-down assays

The DNA encoding the cytoplasmic domain of chB6.1 was amplified by PCR and ligated in-frame with the glutathione-S-transferase coding sequence in the pGEX-6P-3 vector (Amersham Biosciences, Piscataway, NJ). Ligation in-frame was confirmed by sequencing. The vector was transformed into *E. coli* BL-21 cells and fusion proteins induced by addition of 4 μ L of 100 mM isopropyl β-D- thiogalactoside (IPTG). Fusion protein was purified according to the manufacturer’s protocol.

BK3A cells were then split into aliquots of 10^7^ cells. Cell pellets were made up to 0. 5 mL with 10 mM HEPES, pH 7.4, 50 mM β−glycerophosphate, 1% Triton X-100, 10% glycerol, 4 mM EDTA, and 1 mM DTT plus protease inhibitor. 20 μ L of GST Sepharose Slurry was added to each aliquot and was incubated at room temperature for 20 min with gentle agitation. Beads were then removed through centrifugation for 1 min at room temperature, and the supernatant was transferred to clean tubes. GST beads were added into three of the four tubes. The fourth tube was used as a control and consisted of preclear total cell lysates, which did not initially bind to the GST beads. Into each tube, one of the following was added: 10 μ L of purified GST protein, 10 μ L of purified GST-chB6, or 10 μ L of lysis buffer. Tubes were incubated overnight at 4°C with gentle agitation. Next, beads were separated from the supernatant through centrifugation and retained. Beads were washed twice with 250 μl of lysis buffer without protease inhibitor and then resuspended in 10 μ L lysis buffer with inhibitor and 10 μ L protein loading dye. Beads were then boiled for 5 min, quickly cooled on ice for 3 min, and centrifuged for 1 min. Supernatant was then loaded onto a 10% SDS-PAGE gel and then transferred to a nitrocellulose membrane for Western blot as previously described. The antibodies used were Monoclonal Rabbit Anti-Caspase 8 Ab (Cell Signaling Technology, Inc.) in 1:500 and 1:1,000 dilution. Goat-anti-rabbit HRP conjugate used in a 1:2,000 dilution was used in all other blots (Pierce, Rockford, IL.).

### Database searches

Nucleotide sequences of chB6 (accession numbers X92865, X92866, X92867) were used to search the National Center for Biotechnology Information’s (NCBI) Expressed Sequence Tag database nucleotide BLAST (nblast) (18, 19) search engine. EST retrievals were translated from nucleotide sequence to amino acid sequence using the Expert Protein Analysis System (www.expasy.org) provided by the Swiss Institute of Bioinformatics. ESTs identified in this search were individually analyzed by alignment to identify both amino acid similarities with chB6.1 and the potential novel alleles. Alignment figures were produced using the multiple sequence alignment program, Clustal W (20). Alignments with 100% identity to known chB6 were excluded from further analysis. EST clones showing greater than 50% identity to chB6 were analyzed for possible novel allele identification.

EST clones BQ038848, CB016728, and CB017782 were obtained from normalized chicken lymphoid tissue (thymus, bursa, spleen, PBL, and bone marrow, strain of bird not indicated) at the Delaware Institute of Biotechnology in Newark, Delaware, and provided as bacterial cultures in LB broth with ampicillin and 15% glycerol. Clones were streaked onto LB and ampicillin plates at 15-, 30-, and 45- µL aliquots and then incubated over night at 37°C. Single colonies were picked and cultured in liquid broth followed by miniprep and sequencing. EST clone AJ456867 was obtained from a CB strain inbred chicken from the Jean Buerstedde lab, at the Heinrich-Pette-Institute in Hamburg, Germany, and provided as a purified cDNA culture. Purified plasmid DNA was sequenced.

## Results

**Figure 1:**
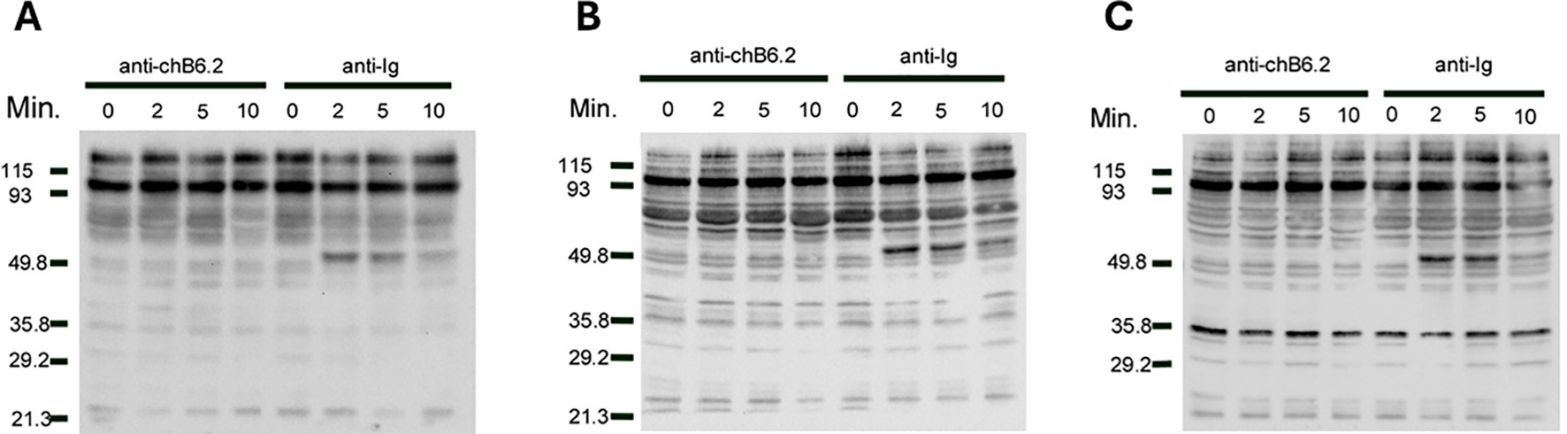
Phosphorylation of intracellular proteins after treatment with anti-chB6.2. DT40 cells were cultured at 5 × 10^6^ cells/mL with antibodies to either chB6.2 or chicken Ig for the times indicated, and then centrifuged at 4°C and resuspended in lysis buffer containing SDS. Volumes of lysate containing 25 mg of protein were separated on SDS-PAGE and blotted to nitrocellulose. Western blots to detect **(A)** phosphoserine, **(B)** phosphothreonine, or **(C)** phosphotyrosine were performed. Detection by chemiluminesence.

Previous work in the laboratory has identified an SH3 binding consensus site (PPMP) at positions 279–282 in the predicted cytoplasmic domain as critical in inducing cell death (16). Mutation of one of the proline residues at this site abrogates the ability of chB6 cross-linking to cause cell death in transfectants. This suggests that one mechanism of signaling from chB6 is via interaction with SH3 domain- containing proteins. To determine if chB6 might also signal via protein phosphorylation, we incubated DT40 cells with antibodies to either chB6 or to chicken Ig heavy and light chains followed by lysis at intervals to determine protein phosphorylation on serine, threonine, and tyrosine residues (**Figure 1**). In each case, we were able to detect increased phosphorylation of cellular proteins within 2 min to 5 min of ligation by anti-Ig antibodies, consistent with other reports of rapid phosphorylation during Ig signaling (21, 22). Conversely, we were not able to detect changes in levels or patterns of phosphorylated proteins after ligation of chB6, arguing that chB6 does not act via protein phosphorylation cascades within the first few minutes.

**Figure 2:**
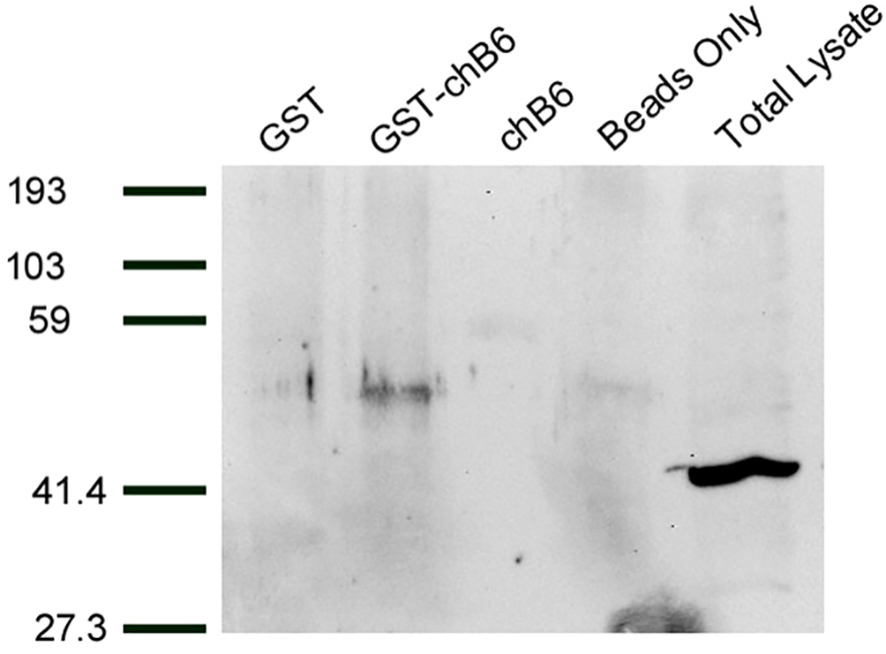
Proteins interacting with GST-chB6 fusion protein. BK3A cells were lysed in buffer containing b-glycerophosphate and Triton X-100. Cell lysates were incubated overnight with sonicates from bacteria producing either GST or GST-chB6. Complexes were precipitated by adding glutathione sepharose beads. Western blots were carried out with anti-caspase 8 serum (Chemi-Con) and visualized by chemiluminescence.

Previous work in the laboratory indicated that caspase 8 is cleaved during chB6-induced apoptosis and was a key step in the cell death process (13, 14). Caspase 8 is also known to be cleaved early in death receptor pathways by being recruited to the cytoplasmic domains of death receptors (23). We hypothesized that caspase 8 would therefore interact with chB6. To test this, the cDNA encoding the cytoplasmic domain of chB6 was fused to glutathione S transferase (GST) in order to perform GST pull down assays. BK3a cell lysates were used because BK3a does not express chB6 (12) so there would be no endogenous chB6 in the lysate to compete with the chB6-GST fusion protein. In multiple experiments (**Figure 2**), we were able to detect anti-Caspase 8 reactive protein in lysates incubated with chB6-GST, but not in lysates incubated with either GST alone or chB6 alone, showing that, at least under the detergent conditions of this assay, chB6 can form a specific interaction with caspase 8.

**Figure 3:**
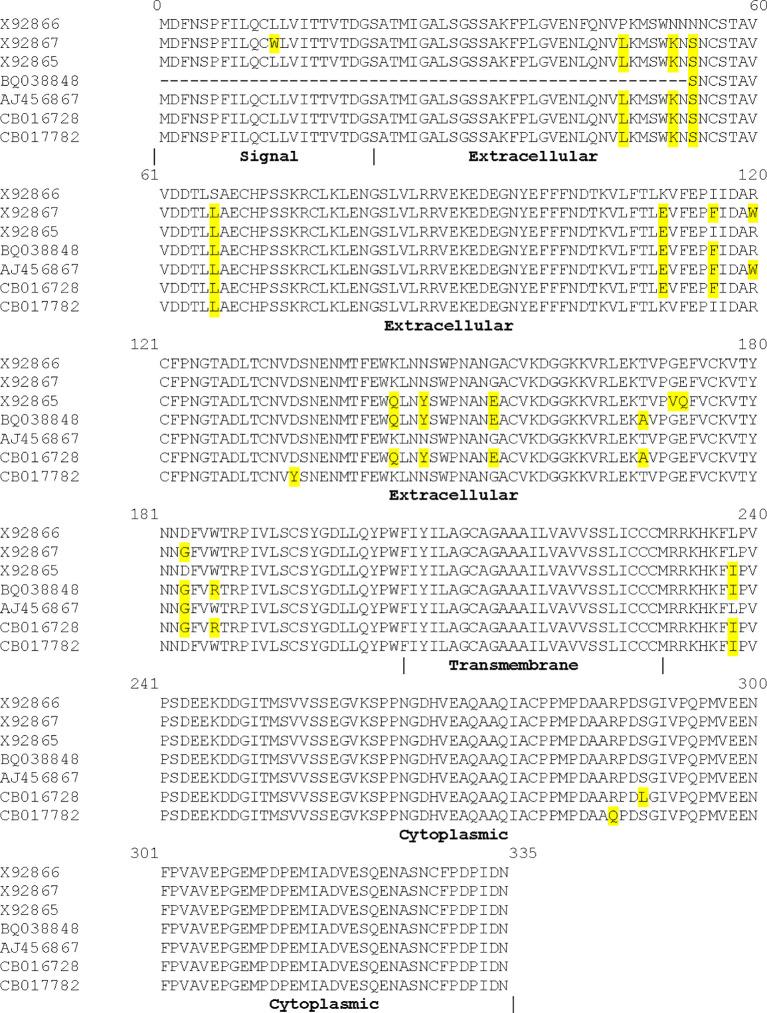
Sequence alignment of novel chB6 alleles identified by EST search. Translated sequences from each of the cDNAs is presented aligned to known chB6 alleles. Differences from X92866 (chB6.1) in highlight. X92867 is chB6.2, and X92865 is chB6.3.

The chB6 protein was predicted to be of novel structure. Subsequent work has shown that the extracellular domain is likely composed of two Ig- like domains, placing it within the CD2/SLAM (Signaling Lymphocyte Activation Molecule) family (15). However, the reported homology with SLAMs is limited to the extracellular domain. Additionally, there were three reported alleles of chB6, all equally distinct from one another and with all changes occurring in the extracellular domain, with the exception of a single isoleucine to leucine substitution at position 238 in the predicted cytoplasmic domain (11). To determine if there were other alleles of chB6, we searched the Expressed Sequence Tag (EST) database using chB6.2 (X92867) as a query (24). Our initial screen identified five EST clones with high homology to chB6. Since EST sequence data are often interpreted by algorithms, we acquired and sequenced each of these on our own. In one case, we determined that the EST data were incorrect and the clone exactly matched the chB6.2 sequence. In the remaining four (accession numbers AJ456867, BQ038848, CB016728, and CB017782), we determined that the reported sequence is correct and that these represent other alleles of chB6 (**Figure 3**, with unique residues highlighted). The new alleles are as distant from each other as from previously reported alleles. In each case, the amino acid changes are present in clusters in the extracellular region of the polypeptide. ChB6.2, chB6.3, AJ456867, CB016728, and CB 017782 all share L46, K51, S53, and L66 but CB017782 does not share E111 with them, instead having K111 in common with X92866. The alleles become more distinct at position 116 where chB.2, BQ038848, AJ456867, and CB016728 all have F whereas the remaining alleles have I. ChB6.2 and AJ456867 both have W at position 120 whereas all other alleles have R. CB017782 is unique in having a Y at position 134. ChB6.3, BQ038848, and CB016728 all share Q144, Y147, and E154. BQ038848 and CB016728 share A169 and R186, but not the previously mentioned W at position 120. ChB6.3 is unique in having a VQ substitution at 172–173. ChB6.2, BQ038848, AJ456867, and CB016728 all share a G at position 183. The only changes in the intracellular domain are a conservative L to I change at position 238 in four of the alleles, a change of S to L at position 289 in CB016728, and a change of R to Q at position 286 of CB017782. In all clones, we examined the palmitoylation signal and the PPMP motif at position 279–282, and the presence of prolines and acids previously noted in the cytoplasmic domains of chB6.1, 6.2, and 6.3 is maintained.

**Figure 4:**
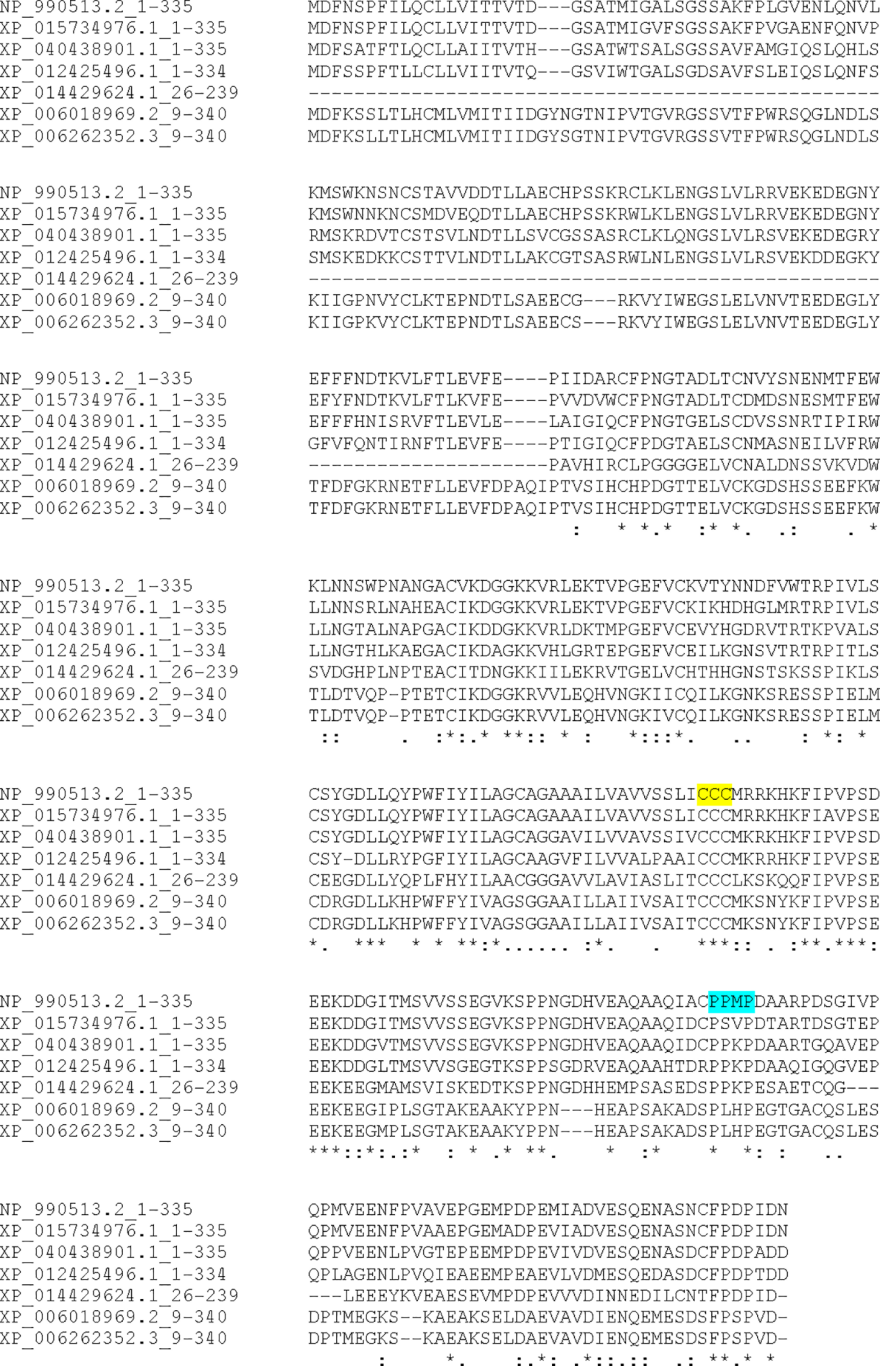
Sequence alignment of selected chB6 orthologs Identified by PSI-BLAST. Amino acid sequences were aligned by CLUSTAL-W. NP-990513.2 is chB6.1, XP_015734976.1 is *Coturnix japonica*, XP_040438901.1 is *Falco naumanni*, XP_012425496.1 is *Taeniopygia guttata*, XP_014429624.1 is *Pelodiscus sinensis*, XP_006018969.2 is *Alligator sinensis*, and XP_006262352.3 is *Alligator mississippiensis*. The conserved CCC palmitoylation site is highlighted in yellow, and the conserved PXXP SH3 binding site is highlighted in turquoise.

Searches of chB6 using the FUGUE sequence-structure homology server supported the presence of two extracellular Ig-like domains in chB6, placing it within the larger SLAM family of cell signaling molecules (15). While that earlier work reported using the PSI-BLAST search and the identification of putative orthologs, the data were not presented. Given our interest in the signaling properties of the cytoplasmic domain, we returned to that work with a focus on the cytoplasmic domain. PSI-BLAST (18, 19) searching using the chB6.1- translated sequence yielded many likely orthologs across genera of birds as well as likely orthologs among reptiles, notably crocodilians and turtles all with identity scores above 37% over the length of the query sequence. While most of these are listed as either hypothetical protein or uncharacterized protein, a number, particularly among the more distant orthologs, are identified as potential CD2 orthologs. CD2 has a proline- rich cytoplasmic domain that is critical for signaling and, due to its two Ig-like domains, is the prototypical member of the larger CD2/SLAM family of genes (25, 26). Example orthologs are presented **Figure 4**. The sequence listed for *P. sinensis* appears to be truncated and missing part of the extracellular domain. Clustal W alignment of these sequences (**Figure 4**) shows absolute conservation of the CCC motif for palmitoylation (highlighted in yellow), retention of the proline- rich and acidic nature of the cytoplasmic domain overall, and conservation of the SH3 motif (highlighted in turquoise). The SH3 motif was conserved among crocodilian and turtle sequences but was not conserved among the snake and lizard sequences we examined. Conservation of these motifs across such a wide evolutionary distance strengthens the argument that they are critical to important biological functions.

## Discussion

The chicken is a foundational model of immunity, and, despite work increasingly shifting to human and mouse models, it is still relevant to our understanding of vertebrate immunity (1, 2, 27). By viewing immunity outside of the mammalian paradigm, we can gain novel insights into more fundamental mechanisms in immunity.

ChB6 initially presented as a molecule of novel structure, and there were no clear mammalian homologs (11). As molecular genetic resources have increased, we have been able to glean new insights into chB6 function. Since chB6 was initially discovered as an alloantigen, it is perhaps not so surprising to discover more alleles as we report here. The CD2/SLAM family is noted for rapid diversification and highly polymorphic alleles (26, 28, 29). However, the clustering of polymorphisms to distinct areas in the extracellular domain may offer insights into putative extracellular ligands to chB6 and suggest new experimental directions. The clustering of chB6 with BCR in lipid rafts strongly suggests a signaling function tied to B- cell activation (15). Evidence placing chB6 within the CD2/SLAM family alludes to a role in “tuning” immune signals, and CD2 family members are known to impact signal strength and duration in a number of contexts (26, 29–32). SLAM genes are highly polymorphic in both human and mice with some polymorphisms associated with autoimmune disease (26, 30–33)). ChB6 has been correlated with enhanced immune responses to viral challenge in a number of studies (34–37). A future experimental goal could be determining if the distinct alleles have impacts on signaling in B cells. Much work on cell signaling has been done in the DT40 system, which co-expresses chB6.1 and chB6.2, so baseline data with those alleles are well established (15). This is because SLAM molecule- mediated homotypic adhesion variation in the extracellular domain could impact the stability of cell:cell adhesion.

The conservation of the intracellular domain of chB6 presents a different set of questions. Since this part of the molecule is presumably responsible for transmitting signals to intracellular machinery, it would likely be more conserved, and that is indeed what the evidence here supports. Among avian orthologs, conservation of the amino acid sequence is quite high, with most species having greater that 60% identity throughout the entire peptide sequence (data not shown). More striking is the conversation among the cytoplasmic domain (**Figure 4**), with conservation of seemingly important motifs such as the palmitoylation signal and the PXXP SH3 binging site (15). There is also notable conservation of other features, such as the positioning of proline residues and the overall content of acidic amino acids. Even among crocodilians and turtles, where amino acid identity scores are understandably lower, the palmitoylation site is conserved. The SH3 binding site is also conserved with at least a similar location relative to the palmitoylation site. This level of similarity between some reptiles and birds is notable. It is also notable that these motifs are not shared with lizards and snakes.

The connection of chB6 with presumptive CD2-like molecules as orthologs in this work is also notable. CD2 is sometimes considered the initial member of the CD2/SLAM family (26). However, CD2 does not signal via SLAM- associated proteins (SAP) but uses a variety of mechanisms connected to its proline-rich cytoplasmic domain (25, 26). CD2 can mediate a wide variety of functions in T cells and is closely associated with the T- cell receptor in the immunological synapse, possibly mirroring the close association of chB6 with the BCR. CD2 has been shown to mediate a range of effects in T cells and NK cells, depending on the signaling context, including apoptosis in some cases and enhanced proliferation in others (25, 26, 30–33). This range of functions is notable in the SLAM family as they are involved in tuning immune responses. Additionally, chB6 is located adjacent to CD2 on chicken chromosome 1, mirroring the chromosomal clustering of CD2 family members in mammals (15).

The work presented here advances our understanding of chB6 and offers new directions toward understanding this enigmatic molecule. It also underscores the vitality of the chicken as a model to understand immunity.

## Data Availability

Publicly available datasets were analyzed in this study. This data can be found here: NCBI Accession numbers BQ038848, CB016728, CB017782 AJ456867.
